# Supplementary material for: Production of a hybrid capacitive storage device via hydrogen gas and carbon electrodes coupling
Source: Nat Commun. 2022 May 19;13:2805. doi: 10.1038/s41467-022-30450-0 (PMC9120448; doi:10.1038/s41467-022-30450-0)
Supplement: Supplementary file 1 — Supplementary Information [file 41467_2022_30450_MOESM1_ESM.pdf]

Supplementary Information

Production of a hybrid capacitive storage device via hydrogen gas and carbon electrodes coupling

Zhengxin Zhu[1,2], Zaichun Liu[1,2], Yichen Yin[1], Yuan Yuan[1], Yahan Meng[1], Taoli Jiang[1], Qia Peng[1], Weiping Wang[1], Wei Chen[1,*]

[1]Department of Applied Chemistry, School of Chemistry and Materials Science, Hefei National Research Center for Physical Sciences at the Microscale, University of Science and Technology of China, Hefei, Anhui 230026, China

[2]These authors contributed equally to this work.

[*]Corresponding author. E-mail address: weichen1@ustc.edu.cn (W. Chen)

Supplementary Figures

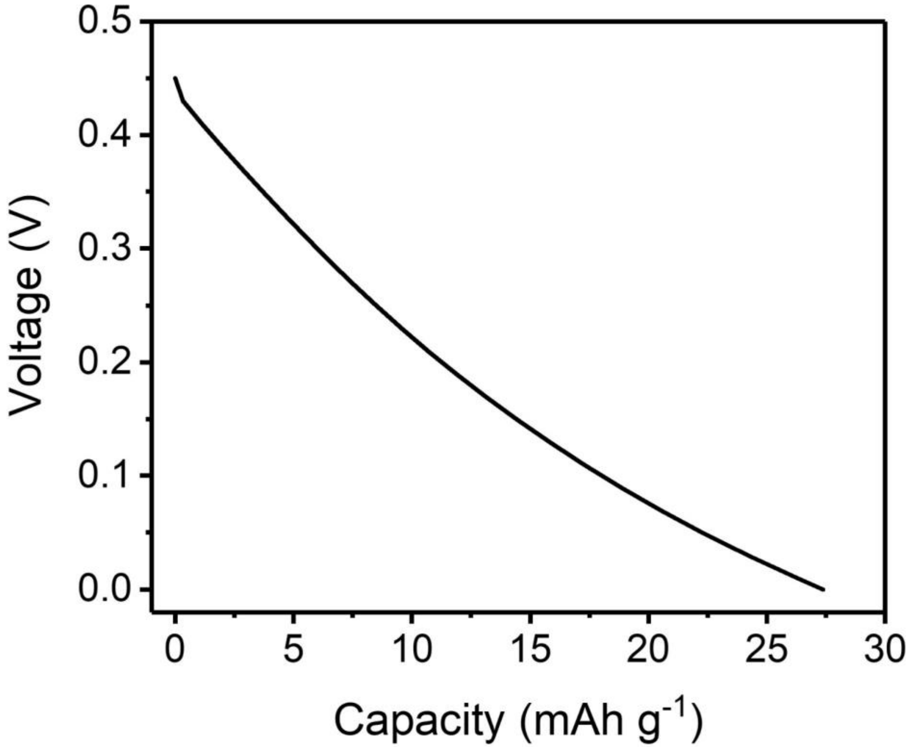

Supplementary Figure 1 | The initial discharge curve of EHGC at a specific current of 1 A g^{-1} in an acidic electrolyte of 9 M H_3PO_4. The electrochemical measurement was carried out at room temperature (25 °C).

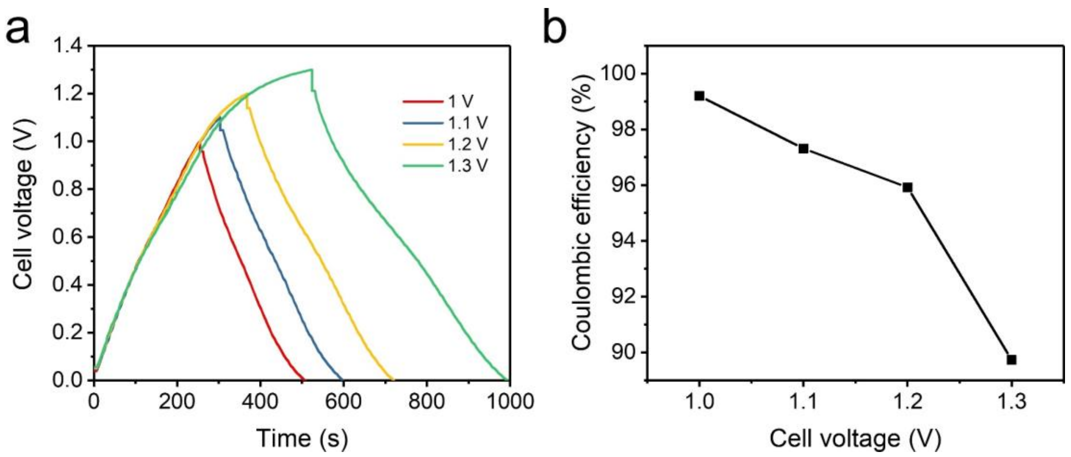

Supplementary Figure 2 | (**a**) Charge/discharge curves and (**b**) corresponding Coulombic efficiency of EHGC at different voltage ranges at a specific current of 1 A g^{-1} in an acidic electrolyte of 9 M H_3PO_4. The electrochemical measurements were carried out at room temperature (25 °C).

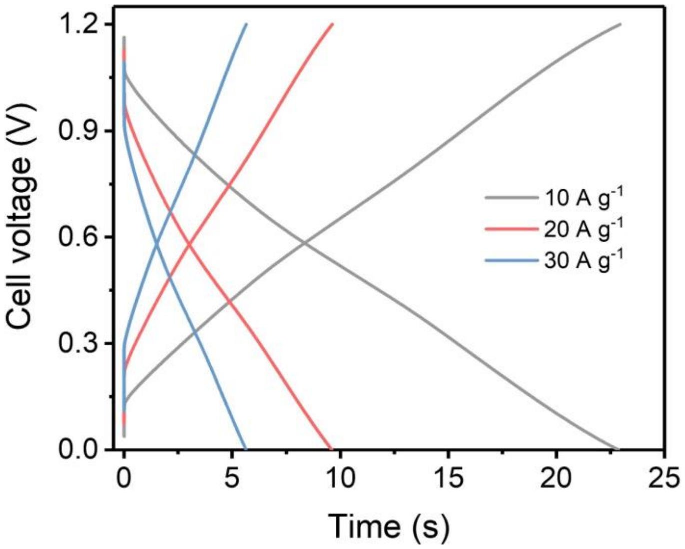

Supplementary Figure 3 | Charge/discharge curves of EHGC at a voltage range of 0-1.2 V at different specific currents in an acidic electrolyte of 9 M H_3PO_4. The electrochemical measurements were carried out at room temperature (25 °C).

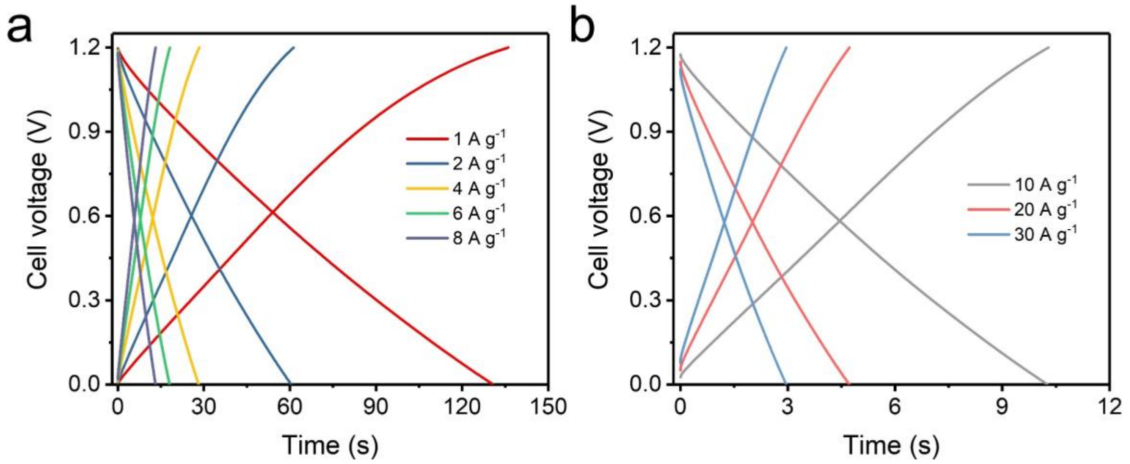

Supplementary Figure 4 | Charge/discharge curves of EDLC at a voltage range of 0-1.2 V at different specific currents in an acidic electrolyte of 9 M H_3PO_4. The electrochemical measurements were carried out at room temperature (25 °C).

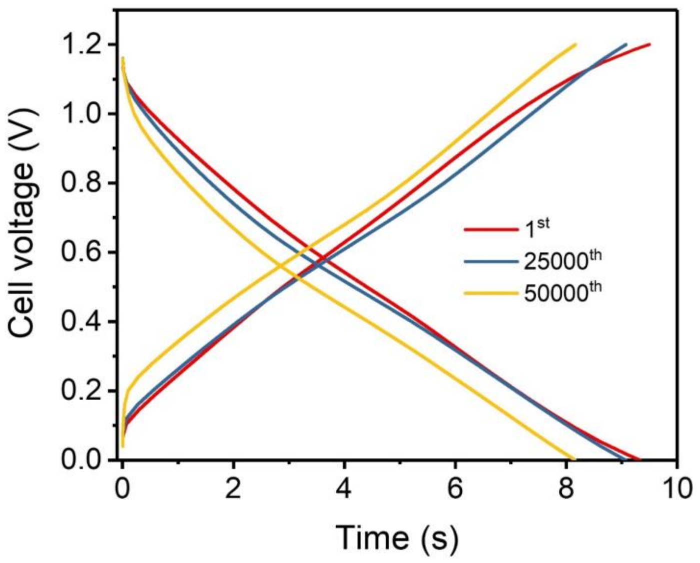

Supplementary Figure 5 | Charge/discharge curves of EHGC at different cycles at 20 A g^{-1} in an acidic electrolyte of 9 M H_3PO_4. The electrochemical measurements were carried out at room temperature (25 °C).

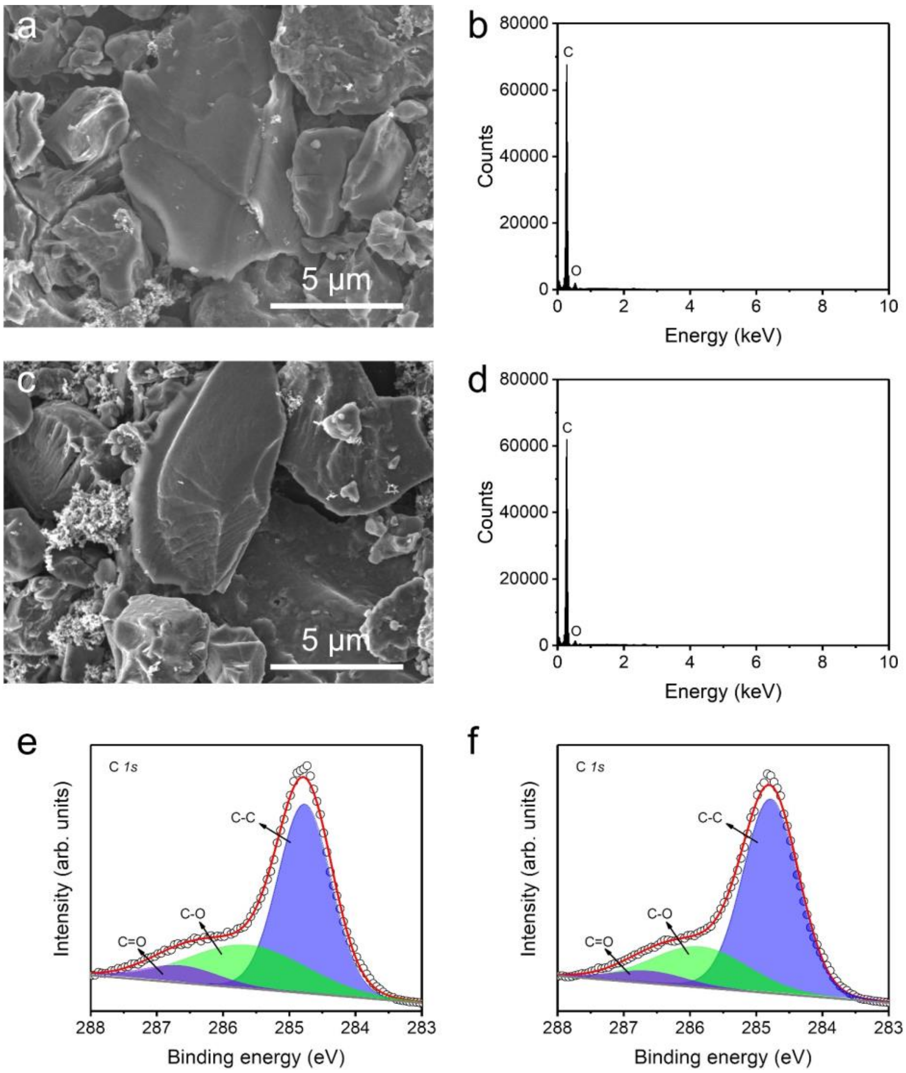

Supplementary Figure 6 | (**a**) Ex situ SEM image and (**b**) EDX analysis of fresh AC electrode. (**c**) Ex situ SEM image and (**d**) EDX analysis of AC electrode after 10,000 cycles at a specific current of 1 A g^{-1} in an acidic electrolyte of 9 M H_3PO_4. Ex situ XPS spectra of (**e**) fresh AC electrode and (**f**) AC electrode after 10,000 cycles in an acidic electrolyte of 9 M H_3PO_4. The electrochemical measurements were carried out at room temperature (25 °C).

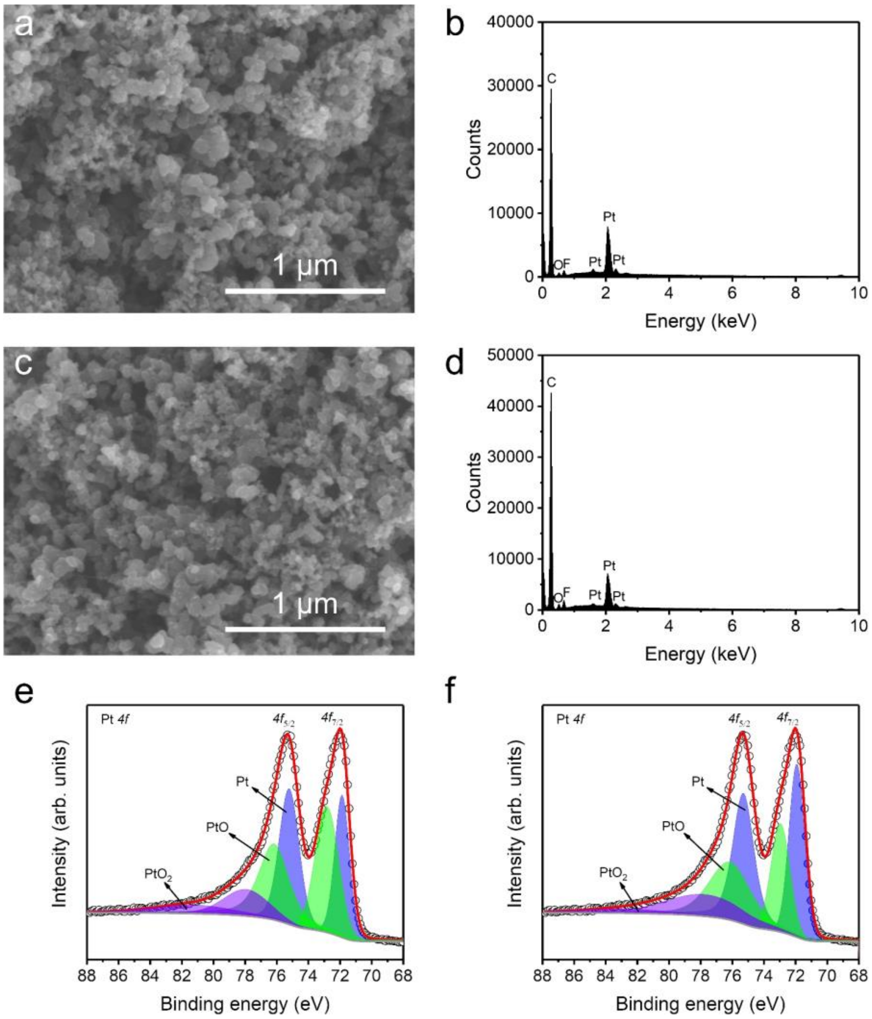

Supplementary Figure 7 | (**a**) Ex situ SEM image and (**b**) EDX analysis of fresh Pt/C electrode. (**c**) Ex situ SEM image and (**d**) EDX analysis of Pt/C electrode after 10,000 cycles at a specific current of 1 A g^{-1} in an acidic electrolyte of 9 M H_3PO_4. Ex situ XPS spectra of (**e**) fresh Pt/C electrode and (**f**) Pt/C electrode after 10,000 cycles in an acidic electrolyte of 9 M H_3PO_4. The electrochemical measurements were carried out at room temperature (25 °C).

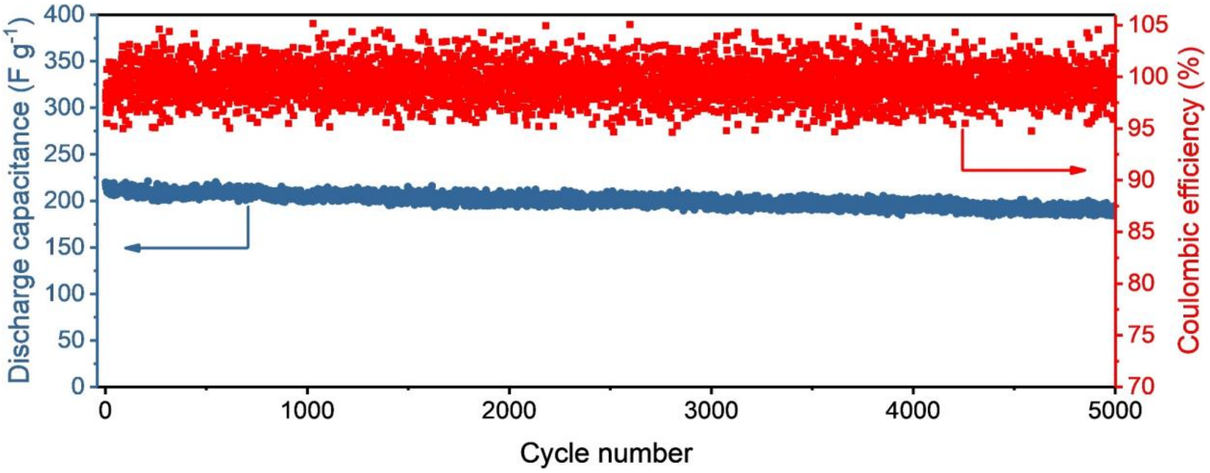

Supplementary Figure 8 | Cycling performance of EHGC at a specific current of 4 A g^{-1} in an acidic electrolyte of 9 M H_3PO_4. The electrochemical measurements were carried out at room temperature (25 °C).

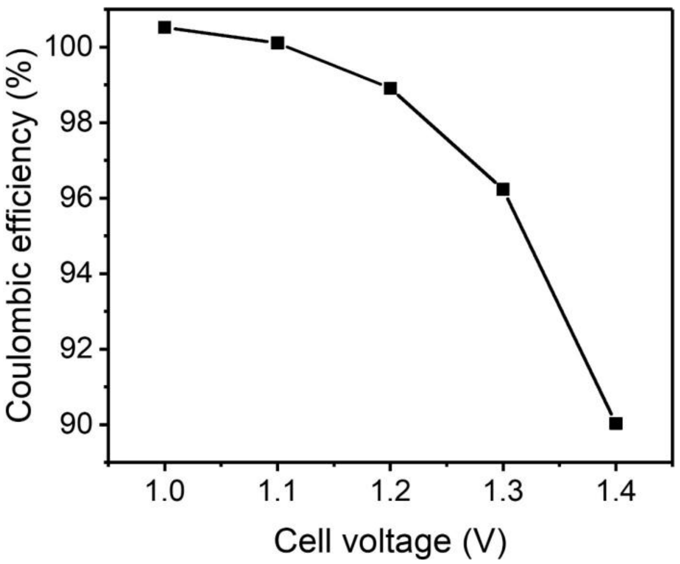

Supplementary Figure 9 | (a) Corresponding Coulombic efficiency of EHGC at different voltage ranges at a specific current of 1 A g^{-1} in a neutral electrolyte of 1 M PBS. The electrochemical measurements were carried out at room temperature (25 ℃).

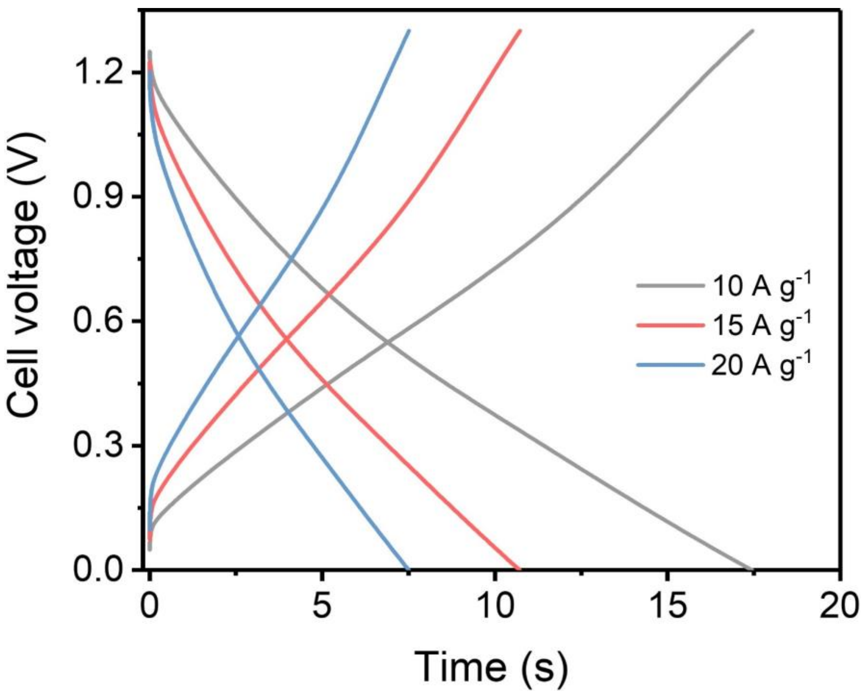

Supplementary Figure 10 | Charge/discharge curves of EHGC at a voltage range of 0-1.3 V at different specific currents in a neutral electrolyte of 1 M PBS. The electrochemical measurements were carried out at room temperature (25 °C).

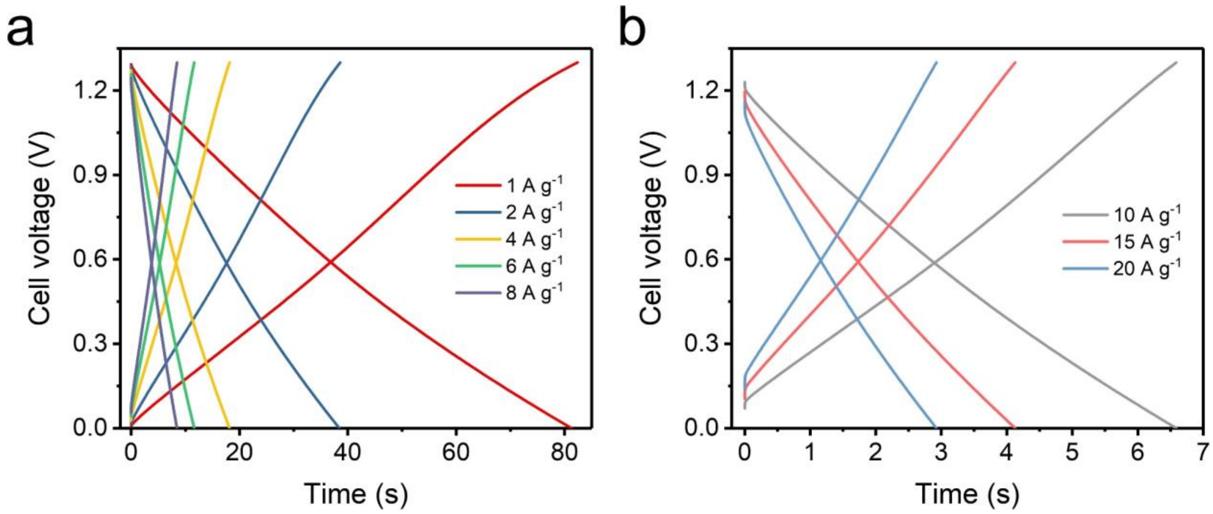

Supplementary Figure 11 | Charge/discharge curves of EDLC at a voltage range of 0-1.3 V at different specific currents in a neutral electrolyte of 1 M PBS. The electrochemical measurements were carried out at room temperature (25 °C).

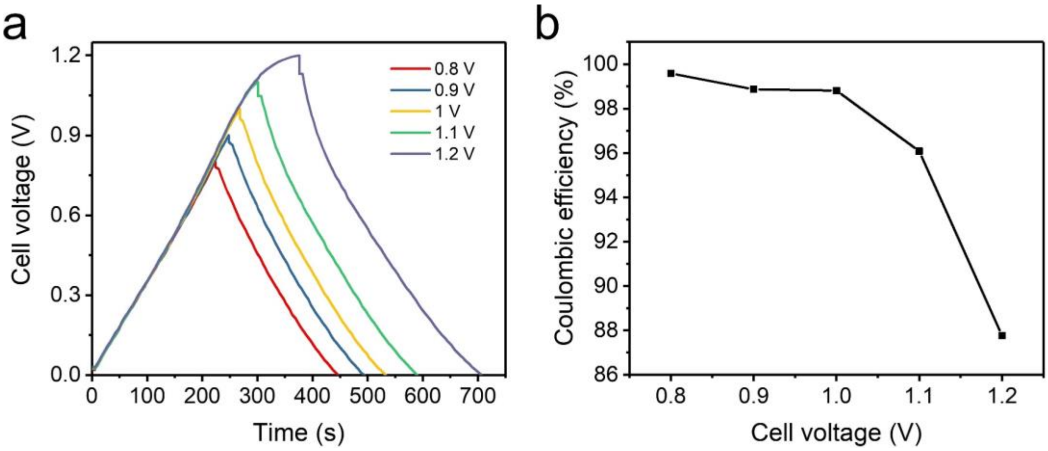

Supplementary Figure 12 | (**a**) Charge/discharge curves and (**b**) corresponding Coulombic efficiency at different voltage ranges at a specific current of 1 A g^{-1} in an alkaline electrolyte of 2 M KOH. The electrochemical measurements were carried out at room temperature (25 °C).

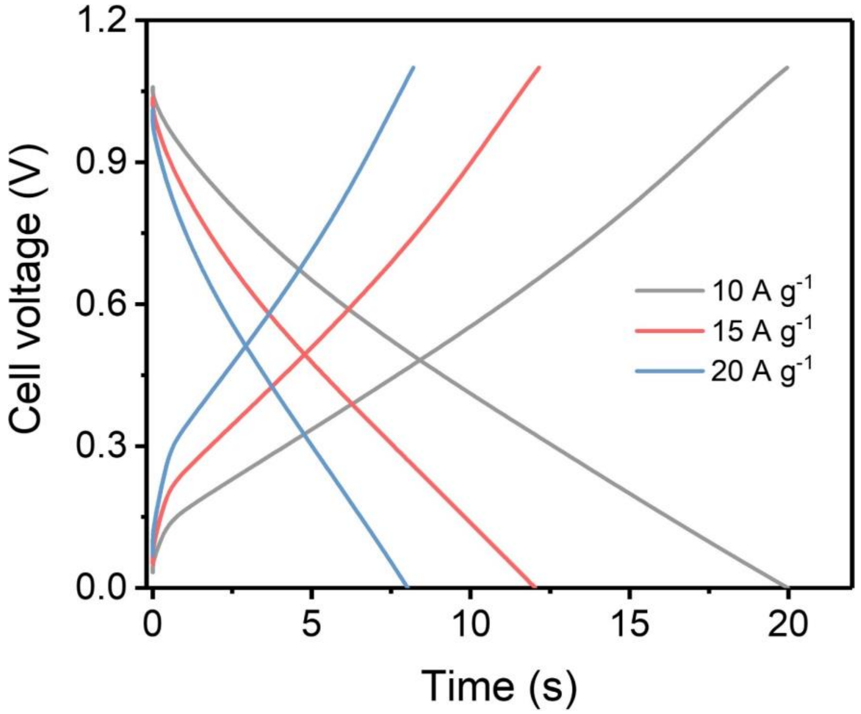

Supplementary Figure 13 | Charge/discharge curves of EHGC at a voltage range of 0-1.1 V at different specific currents in an alkaline electrolyte of 2 M KOH. The electrochemical measurements were carried out at room temperature (25 °C).

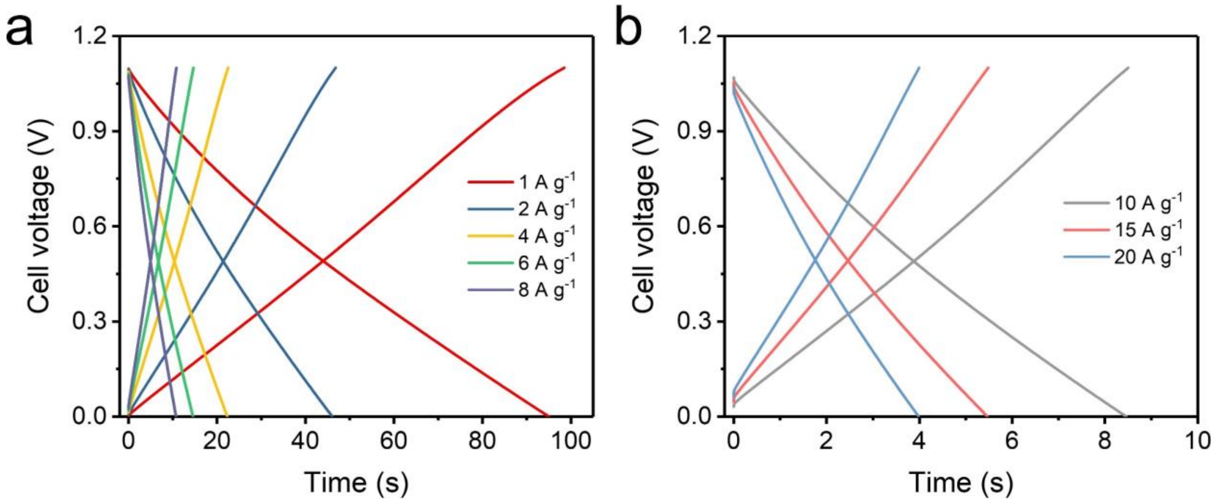

Supplementary Figure 14 | Charge/discharge curves of EDLC at a voltage range of 0-1.1 V at different specific currents in an alkaline electrolyte of 2 M KOH. The electrochemical measurements were carried out at room temperature (25 °C).

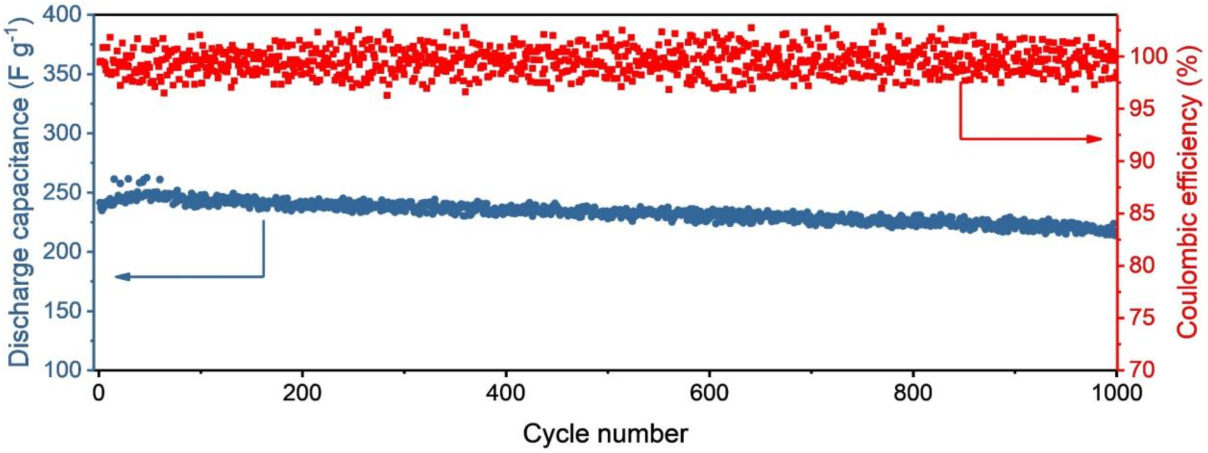

Supplementary Figure 15 | Cycling performance of EHGC at a specific current of 20 A g^{-1} at 60 °C in an acidic electrolyte of 9 M H_3PO_4.

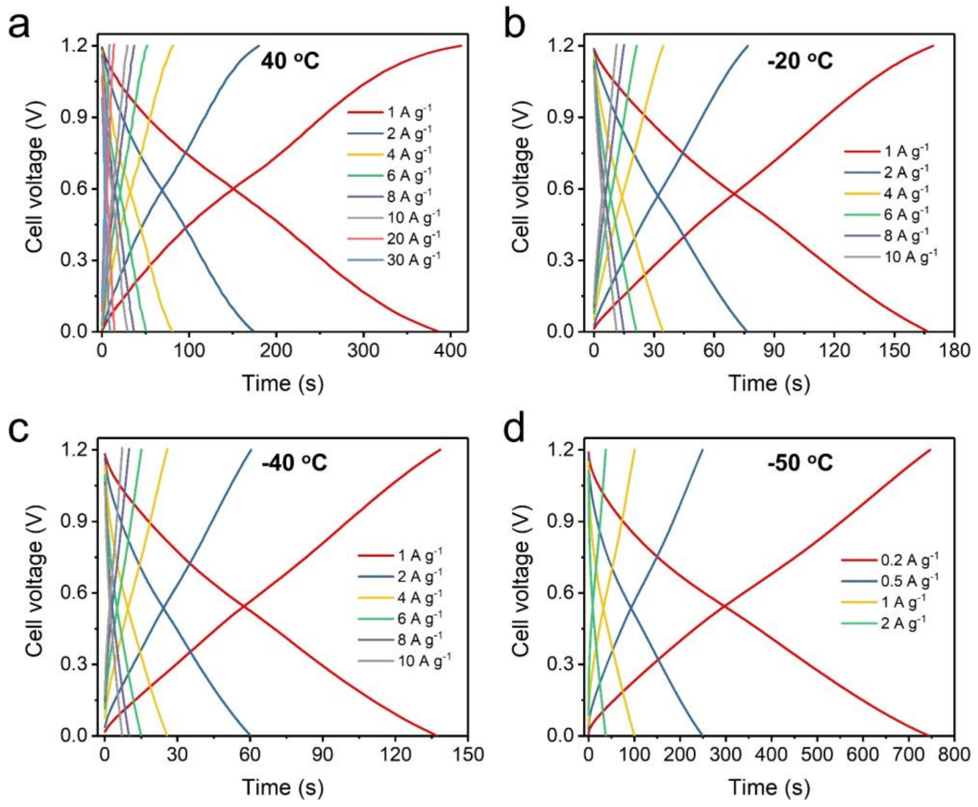

Supplementary Figure 16 | Charge/discharge curves of EHGC at a voltage range of 0-1.2 V with different specific currents at **a** 40 °C, **b** −20 °C, **c** −40 °C, and **d** −50 °C in an acidic electrolyte of 9 M H_3PO_4.

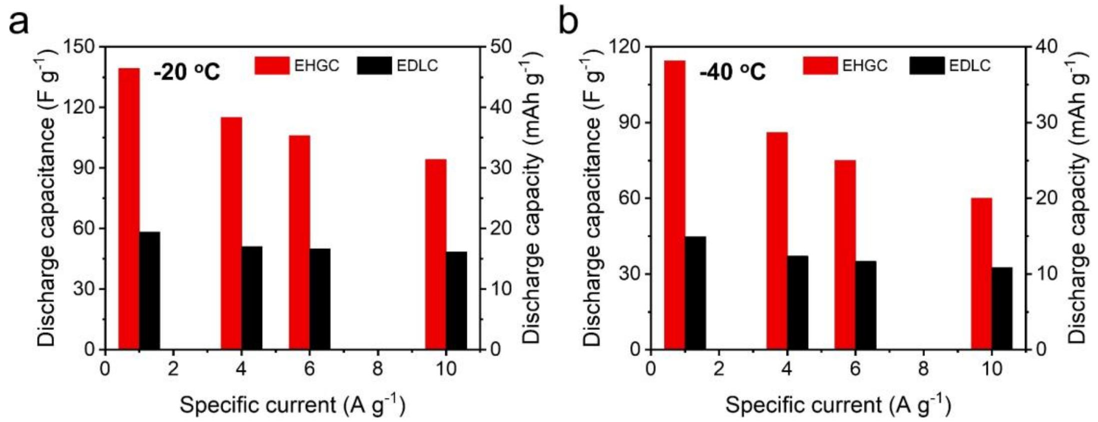

Supplementary Figure 17 | Specific capacitance and specific capacity as a function of the specific current for EHGC and EDLC at (**a**) −20 °C and (**b**) −40 °C in an acidic electrolyte of 9 M H_3PO_4.

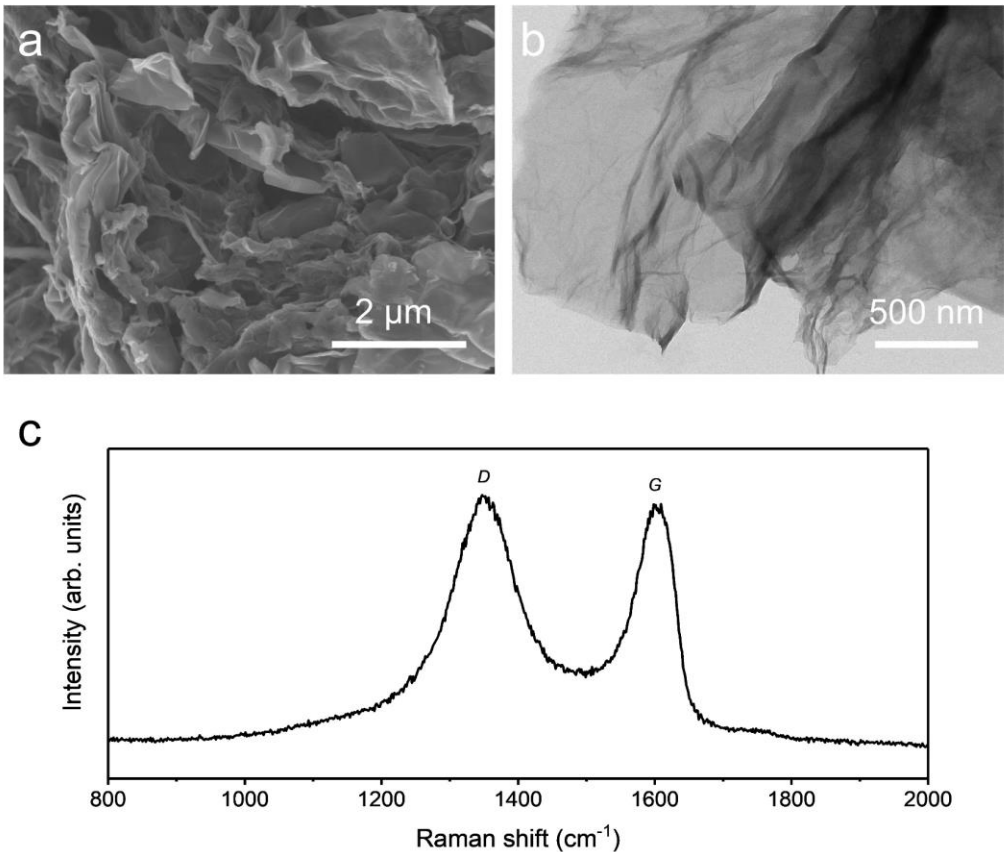

Supplementary Figure 18 | (**a**) SEM image, (**b**) TEM image, and (**c**) Raman spectrum of fresh rGO electrode.

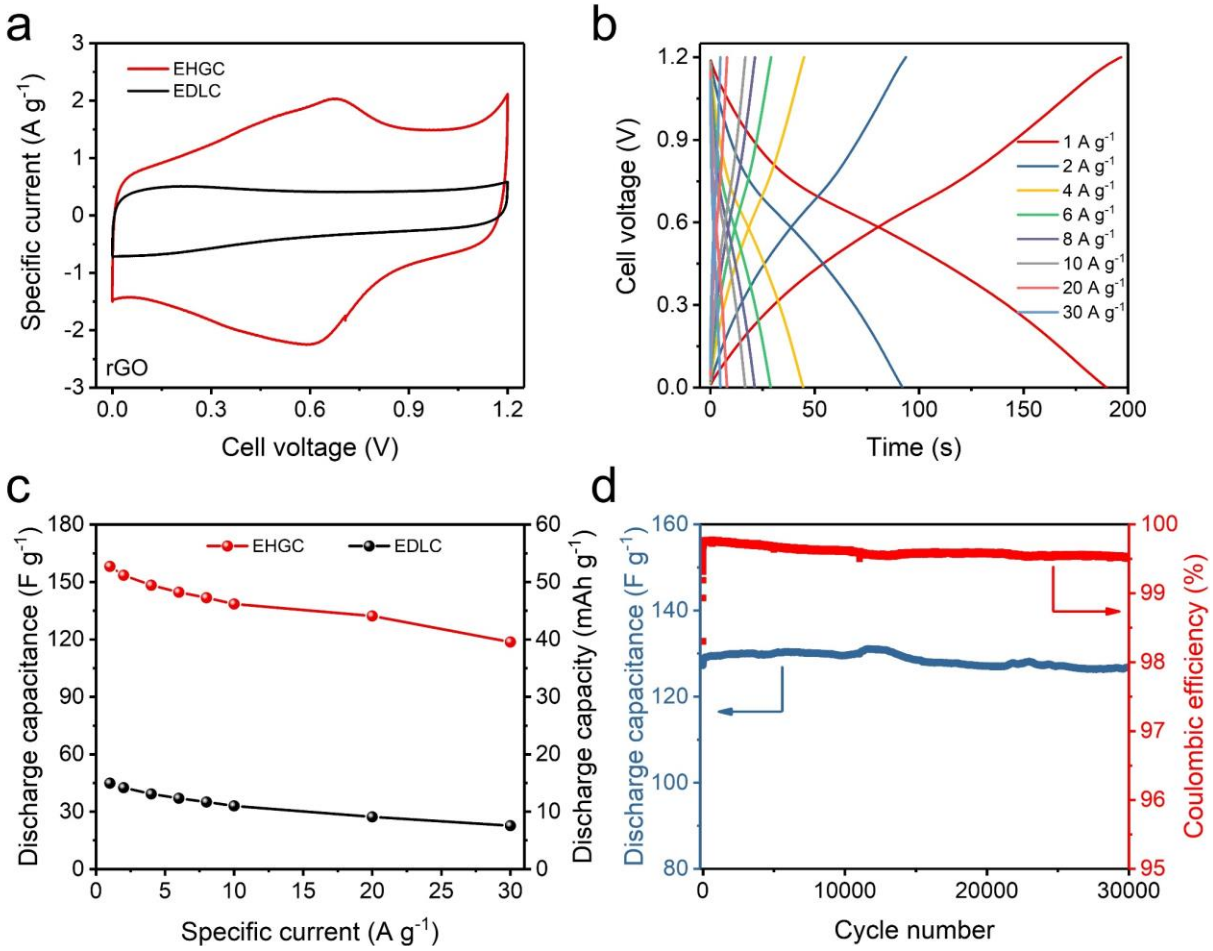

Supplementary Figure 19 | Electrochemical performance of the EHGC in acidic electrolyte by using rGO electrode. (**a**) CV curves of EHGC and EDLC at the scan rate of 10 mV s^{-1}. (**b**) Charge/discharge curves at a voltage range of 0-1.2 V at different specific currents. (**c**) Specific capacitance and specific capacity as a function of the specific current for EHGC and EDLC. (**d**) Cycling performance at a specific current of 20 A g^{-1}. The electrochemical measurements of the EHGCs were carried out at room temperature (25 °C) in an acidic electrolyte of 9 M H_3PO_4. EDLC: electric double-layer capacitor. EHGC: electrocatalytic hydrogen gas capacitor.

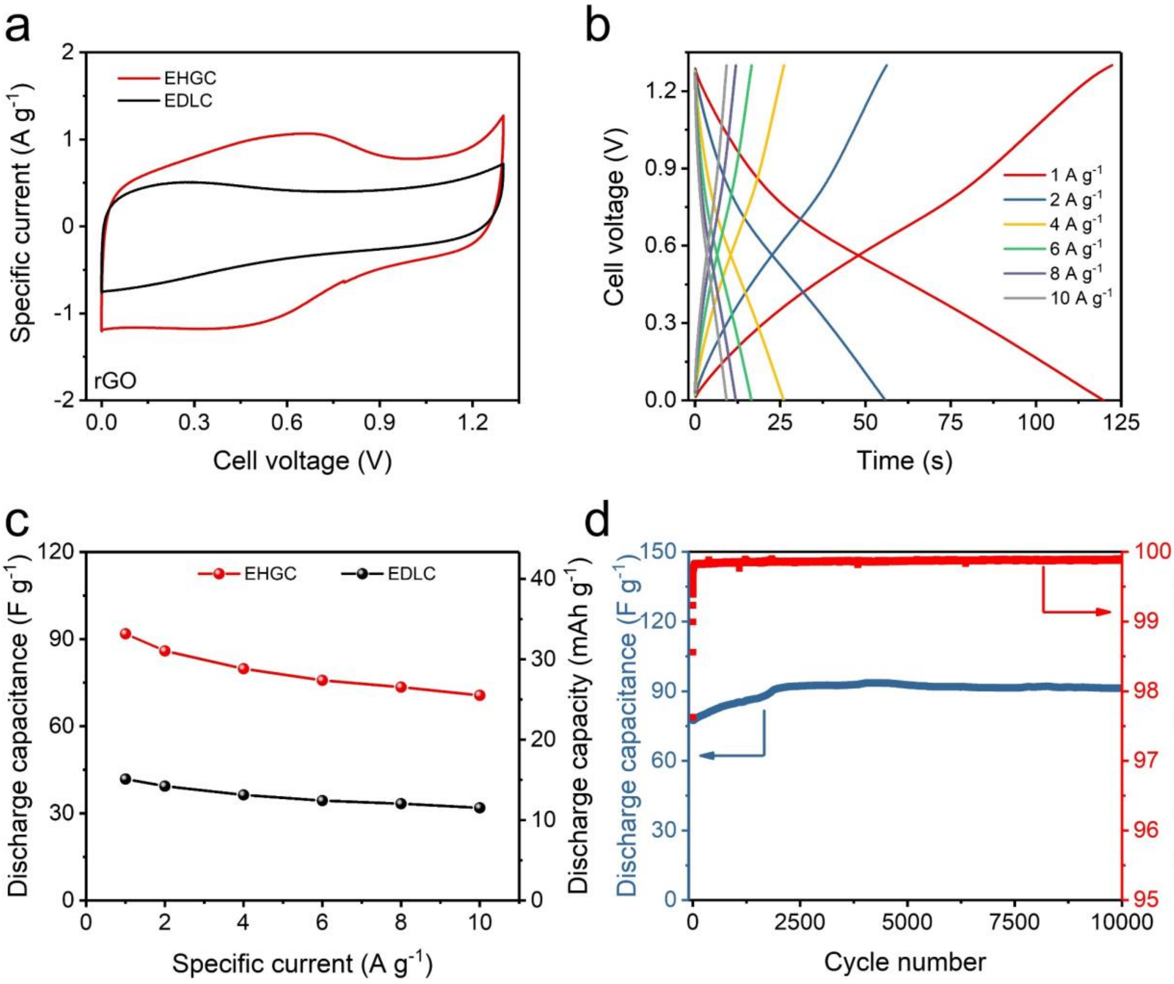

Supplementary Figure 20 | Electrochemical performance of the EHGC in neutral electrolyte by using rGO electrode. (**a**) CV curves of EHGC and EDLC at the scan rate of 10 mV s^{-1}. (**b**) Charge/discharge curves at a voltage range of 0-1.3 V at different specific currents. (**c**) Specific capacitance and specific capacity as a function of the specific current for EHGC and EDLC. (**d**) Cycling performance at a specific current of 6 A g^{-1}. The electrochemical measurements of the EHGCs were carried out at room temperature (25 ℃) in a neutral electrolyte of 1 M PBS. EDLC: electric double-layer capacitor. EHGC: electrocatalytic hydrogen gas capacitor.

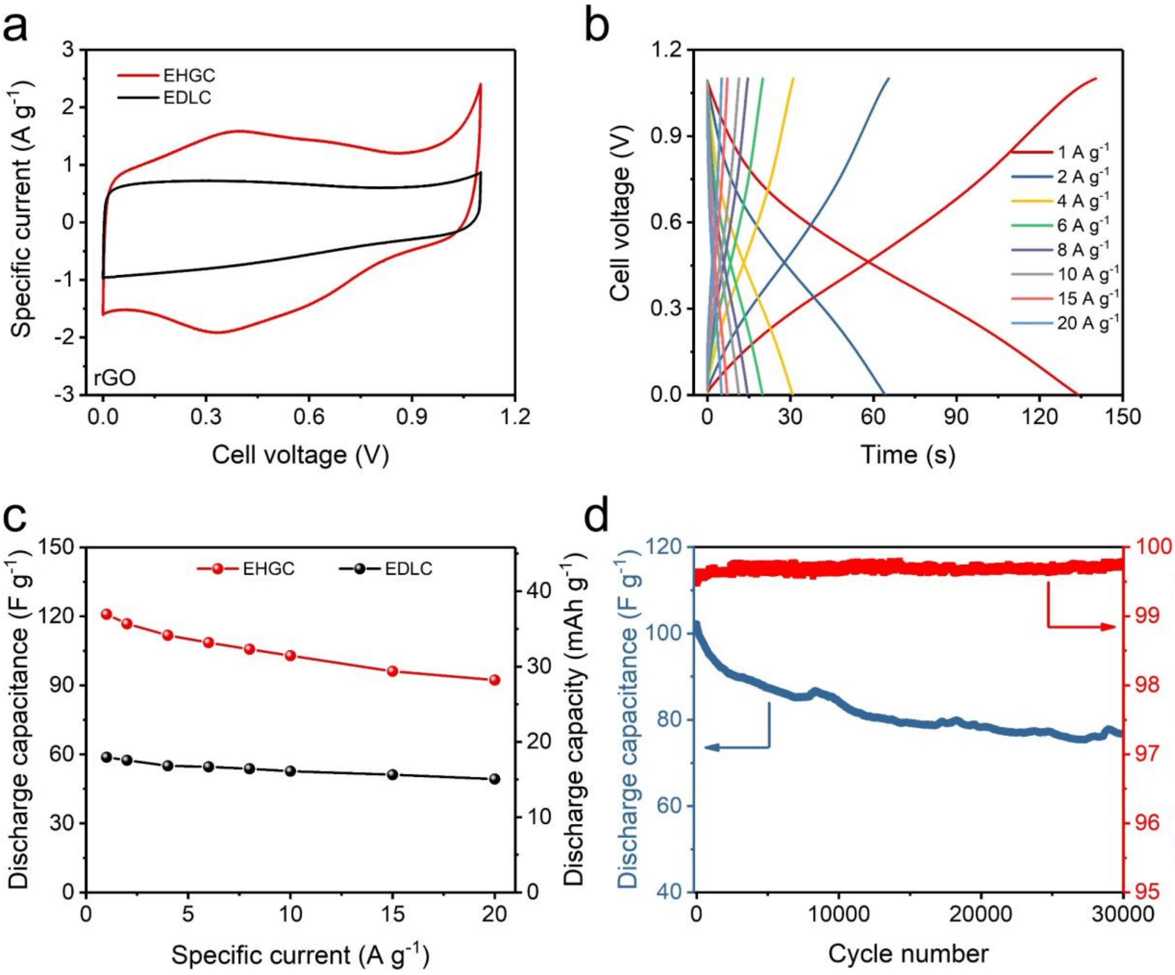

Supplementary Figure 21 | Electrochemical performance of the EHGC in alkaline electrolyte by using rGO electrode. (**a**) CV curves of EHGC and EDLC at the scan rate of 10 mV s^{-1}. (**b**) Charge/discharge curves at a voltage range of 0-1.1 V at different specific currents. (**c**) Specific capacitance and specific capacity as a function of the specific current for EHGC and EDLC. (**d**) Cycling performance at a specific current of 10 A g^{-1}. The electrochemical measurements of the EHGCs were carried out at room temperature (25 ℃) in an alkaline electrolyte of 2 M KOH. EDLC: electric double-layer capacitor. EHGC: electrocatalytic hydrogen gas capacitor.

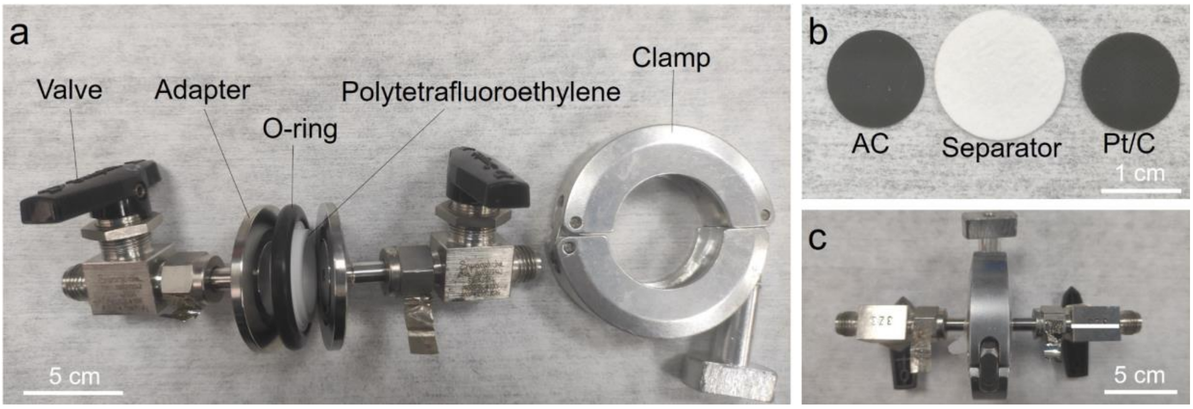

Supplementary Figure 22 | Photographic pictures of the Swagelok-type cell used for the EHGC assembly and testing.

Supplementary Table 1 | Comparison of rate capacitance and cycling retention ratio of the EHGC with reported electrochemical capacitor systems at room temperature (~25 °C).

System	Electrolyte	Capacitance/ Specific current	Mass loading of positive electrode	Retention (%/cycles)	Ref.
**H_2||AC**	**9 M H_3PO_4 (aq)**	**141 F g^{-1}/30 A g^{-1}**	**2 mg cm^{-2}**	**87.5%/50000**	**This work**
Zn||Bio-AC	1 M $Zn(CF_3SO_3)_2$ in acetonitrile	130.9 F g^{-1}/2 A g^{-1}	-	91%/20000	1
Zn||RuO_2·H_2O	2 M $Zn(CF_3SO_3)_2$ (aq)	33.8 F g^{-1}/20 A g^{-1}	2.5-3 mg cm^{-2}	87.5%/10000	2
TiC||TiC	3 M KOH (aq)	60 F g^{-1}/40 A g^{-1}	-	80.5%/20000	3
Zn||MXene	2 M $ZnSO_4$ (aq)	121 F g^{-1}/3 A g^{-1}	-	82.5%/1000	4
rGO||PDI-PDA-rGO	$NaSO_4$ gel (aq)	20 F g^{-1}/5 A g^{-1}	2.5 mg cm^{-2}	96%/10000	5
AC||NiCoP@CoS	3 M KOH (aq)	91.7 F g^{-1}/10 A g^{-1}	-	86.1%/10000	6
Zn||BP	WIS (aq)	46.1 F g^{-1}/6.4 A g^{-1}	0.6-0.8 mg cm^{-2}	46%/9500	7
MXene||RuO_2	1 M H_2SO_4 (aq)	109.1 F g^{-1}/40 A g^{-1}	0.75 mg cm^{-2}	86%/20000	8
POP-TAPP-NTCA||EG/PANI	2 M $ZnSO_4$ (aq)	94 F g^{-1}/1.5 A g^{-1}	1-1.2 mg cm^{-2}	90%/1100	9
Zn||TiN	1 M $ZnSO_4$ (aq)	171.1 F g^{-1}/6.4 A g^{-1}	0.8 mg cm^{-2}	75%/10000	10

Supplementary Table 2 | Comparison of low-temperature capacitance of the EHGC with reported metal-ion capacitors.

System	Electrolyte	Capacitance/ Specific current	Mass loading of positive electrode	Testing temperature	Ref.
**H_2||AC**	**9 M H_3PO_4 (aq)**	**139 F g^{-1}/1 A g^{-1}**	**2 mg cm^{-2}**	**-20 °C**	**This work**
**H_2||AC**	**9 M H_3PO_4 (aq)**	**114 F g^{-1}/1 A g^{-1}**	**2 mg cm^{-2}**	**-40 °C**	**This work**
**H_2||AC**	**9 M H_3PO_4 (aq)**	**108 F g^{-1}/0.05 A g^{-1}**	**2 mg cm^{-2}**	**-60 °C**	**This work**
Zn||AC	$ZnSO_4$/H_2O/EG-65 (aq)	83.8 F g^{-1}/0.1 A g^{-1}	7 mg cm^{-2}	-20 °C	11
Zn||AC	$ZnSO_4$/H_2O/EG-65 (aq)	35.8 F g^{-1}/0.1 A g^{-1}	7 mg cm^{-2}	-40 °C	11
Zn||AC	3 m $Zn(ClO_4)_2$ (aq)	112.2 F g^{-1}/0.5 A g^{-1}	1 mg cm^{-2}	-20 °C	12
Zn||AC	3 m $Zn(ClO_4)_2$ (aq)	95.3 F g^{-1}/0.5 A g^{-1}	1 mg cm^{-2}	-40 °C	12
Zn||PC	7.5 m $ZnCl_2$ (aq)	144.4 F g^{-1}/5 A g^{-1}	0.8 mg cm^{-2}	-20 °C	13

Reference list

1. Wang, H., Wang, M. & Tang, Y. A novel zinc-ion hybrid supercapacitor for long-life and low-cost energy storage applications. *Energy Storage Mater.* **13**, 1-7 (2018).
2. Dong, L. et al. High-Power and Ultralong-Life Aqueous Zinc-Ion Hybrid Capacitors Based on Pseudocapacitive Charge Storage. *Nano-Micro Lett.* **11**, 94 (2019).
3. Chen, T., Li, M., Song, S., Kim, P. & Bae, J. Biotemplate preparation of multilayered TiC nanoflakes for high performance symmetric supercapacitor. *Nano Energy* **71**, 104549 (2020).
4. Yang, Q. et al. A Wholly Degradable, Rechargeable Zn-Ti_3C_2 MXene Capacitor with Superior Anti-Self-Discharge Function. *ACS Nano* **13**, 8275-8283 (2019).
5. De Adhikari, A., Morag, A., Seo, J., Kim, J. M. & Jelinek, R. Polydiacetylene-Perylenediimide Supercapacitors. *ChemSusChem* **13**, 3230-3236 (2020).
6. Xu, Z. et al. NiCoP@CoS tree-like core-shell nanoarrays on nickel foam as battery-type electrodes for supercapacitors. *Chem. Eng. J.* **421**, 127871 (2021).
7. Huang, Z. et al. Phosphorene as Cathode Material for High-Voltage, Anti-Self-Discharge Zinc Ion Hybrid Capacitors. *Adv. Energy Mater.* **10**, 2001024 (2020).
8. Jiang, Q., Kurra, N., Alhabeb, M., Gogotsi, Y. & Alshareef, H. N. All Pseudocapacitive MXene-RuO_2 Asymmetric Supercapacitors. *Adv. Energy Mater.* **8**, 1703043 (2018).
9. Cui, F.-Z. et al. Polyarylimide and porphyrin based polymer microspheres for zinc ion hybrid capacitors. *Chem. Eng. J.* **405**, 127038 (2021).
10. Huang, Z. et al. Effects of Anion Carriers on Capacitance and Self-Discharge Behaviors of Zinc Ion Capacitors. *Angew. Chem. Int. Ed.* **60**, 1011-1021 (2021).
11. Li, F. et al. Fabricating low-temperature-tolerant and durable Zn-ion capacitors via modulation of co-solvent molecular interaction and cation solvation. *Sci. China Mater.* **64**, 1609-1620 (2021).
12. Sun, Y. et al. Salty Ice Electrolyte with Superior Ionic Conductivity Towards Low-Temperature Aqueous Zinc Ion Hybrid Capacitors. *Adv. Funct. Mater.* **31**, 2101277 (2021).
13. Wang, C. et al. Toward Flexible Zinc-Ion Hybrid Capacitors with Superhigh Energy Density and Ultralong Cycling Life: The Pivotal Role of $ZnCl_2$ Salt-Based Electrolytes. *Angew. Chem. Int. Ed.* **60**, 990-997 (2021).
